# Notices

**Published:** 1864-08

**Authors:** 


### NOTICES.

THE *Home Journal*, for July second, under the head of "Checking Perspiration," says: "We clip the following from an Edinburgh exchange, and most timely. A suggestion to the wise is enough. The extract merits no small consideration." These are our sentiments precisely, and that is the reason we wrote it two or three years ago for this JOURNAL, in the form of a Health Tract Number 58; we reproduce it in this number, with four others, as specially applicable to the season, and are almost as good as new. This is not the first time our articles have crossed the water twice, and come back to us with a foreign name and paternity. So much the better, as it increases the size of our congregation to two continents instead of one.

The Boston American Tract Society, 28 Cornhill, Boston, and 13 Bible House, New-York, have sent us two beautiful books for "notice," "Progress;" or, the Sequel to *Jerry and his Friends*, by Alice A. Dodge, 346 pp., and "Human Sorrows," by the Countess Agenor De Gasparin, translated from advance sheets, by Mary L. Booth, with a notice of the work, by M. Laboulaye, 279 pp. There are eight chapters on "Oppressions," "Mistakes," "Dejection," "Destruction," "Despair," "Noble Sorrows," and "Death." This is a book for every human creature, for sorrow is the lot of all, and all may find a well-spring of comfort in these pages. One chapter commences thus: "Souls enter life in pairs. On reaching the threshold of this world, they take different roads. Sometimes they find each other; these are privileged souls; oftener they wander at random; each flutter of their pinions separates them; they traverse space fallen and solitary, then disappear; this is the common lot, so say the poets. I know of a more fatal search, that of a man who has elbowed every body, and has never yet encountered himself."

The most execrably printed newspaper that comes to our table is the *State League*, of Syracuse, New-York. We always lay it aside with regret, for the headings of its articles indicate that it is edited with ability and industry. The publisher would, or ought to, make money by getting better paper, and proportionably increasing the subscription price.

MALADIES OF THE MIND.—These are times which try men's souls. The "uncertainty of riches," of property, and all values; the terrible fact that a premature death or painful life-long maimings have entered almost every household in the nation, are well calculated to hang like a pall on the hearts of all. Under these circumstances, we direct the reader's attention to an article written by a friend, entitled, "Christianity Social and Joyous." True religion is the best medicine for a sorrowing heart; behind the seeming gloom of it there is a gladness ineffable, a cloudless sky of glorious sunshine, which scatters doubt, and fear, and despondencies, and so enlivens the soul as to be sufficient of itself in many cases to repel diseases from the body, and invite back physical health; acting upon the same principle is the established fact, that the wounded soldiers of a conquering army recover more rapidly and in larger numbers than do the vanquished. One reflection, one item of "faith," is of itself of priceless value to him who rests on the "sure foundation-stone;" and it is this, "The Judge of all the earth will do right;" He will overrule all things in such a way as to work out the happiest results for the whole human family.

PERSONAL.—A Washington telegram of May 26th, to the Associated Press, in New-York, says: "The Report on Agriculture for the past year, presented to Congress to-day, will contain above forty essays, among which will be another of those sensible essays, by Dr. Hall, of New-York, so admirably designed to enhance the health, comfort and happiness of the rural population, to wit, in the article on 'Farmer's Houses.'"

GRAY HAIRS.—The newspapers say that the hair will return to its natural color, whether light or dark, if it is washed every morning with a mixture of one part of bay rum, three parts of olive oil, and one part brandy. Wish some of our readers would try it, a month or two, and report progress.

"Glorying in the Goad," in the *Atlantic Monthly* for July, by Gail Hamilton, advocates the establishment of Agricultural Colleges; and that citizens' sons should patronize them, and go to farming, and thus get back the constitutional vigor which their fathers brought with them when they came from the country to the town; a new and good idea. The lively and popular authoress pays her respectful respects to our article on the Health of Farmers' Families, written for the reports of the Agricultural Department for 1862, of which Congress ordered the publication of over a hundred thousand copies. The "Report" was made up by the Hon. Isaac Newton, and has met with universal commendation.

